# Tuberculosis Screening before Anti–Hepatitis C Virus Therapy in Prisons

**DOI:** 10.3201/eid1804.111016

**Published:** 2012-04

**Authors:** Sergio Babudieri, Andrea Soddu, Monica Murino, Paola Molicotti, Alberto A. Muredda, Giordano Madeddu, Alessandro G. Fois, Stefania Zanetti, Pietro Pirina, Maria Stella Mura

**Affiliations:** University of Sassari, Sassari, Italy (S. Babudieri, A. Soddu, P. Molicotti, G. Madeddu, M.S. Mura, S. Zanetti, A.G. Fois, P. Pirina);; Ministry of Justice, Sassari (M. Murino, A.A. Muredda)

**Keywords:** tuberculosis and other mycobacteria, purified protein derivative, PPD, hepatitis C virus, HCV, prisons, pegylated interferon, viruses

**To the Editor**: Prisons represent a crucial setting for tuberculosis (TB) control. Worldwide, reported TB rates for correctional system populations have been 10–100× higher than rates for the local civilian populations, and TB outbreaks with a high number of TB multidrug-resistant cases have been documented (*1**,**2*). Prisons are known as social and sanitary pathology reservoirs in which TB is often associated with chronic infectious diseases caused by HIV, hepatitis B virus (HBV), or hepatitis C virus (HCV) (*2*).

HCV prevalence among inmates is 30%–40% (range 2%–58%), which is higher than that in the general population and is related to injection drug use (*3*). For these reasons, effective anti-HCV therapeutic approaches are recommended by national and international guidelines for decreasing illness, death rates, and reservoirs of infection in prisons (*4**,**5*).

The standard of care for patients with chronic hepatitis C infection is represented by pegylated interferon-α (Peg-IFN) and ribavirin. These drugs determine complex antiviral, immunomodulatory, and antiproliferative actions, which can cause serious side effects such as leukopenia/neutropenia and alterations in the cytokine network (*3*). Although severe cellular immunodeficiency can often facilitate the development of many infections, only 4 clinical cases of TB in patients undergoing HCV antiviral therapy have been described in the literature (*6**–**8*), and only 1 of these was clearly described as a TB reactivation (*7*).

We describe a case of pulmonary TB reactivation during therapy with Peg-IFN and ribavirin in a 44-year-old white male inmate, affected by genotype 1b/4a chronic hepatitis C. After prison admission in 2009, he underwent routine screening tests for infectious diseases, which indicated HCV antibody, HBV surface antibody, HBV core IgG antibody, and tuberculin skin test positivity. Results of chest radiograph and HIV screening were negative.

His previous history involved injection drug use, smoking, and alcohol consumption. Anti-HCV therapy of directly observed administration of Peg-INF α-2a (180 µg/wk) and ribavirin (1,200 mg/d) was started. During therapy, the patient had only mild musculoskeletal pain and temporary irritability. During the 12th week of treatment, HCV-RNA decreased by 1 log_10_; therefore, the ribavirin dose was increased to 1,600 mg per day. Even after the therapy modification, no virologic suppression was found. Although during the 33rd week of therapy the patient had weakness, cough, and 2 episodes of hemoptysis, the results of a physical examination were unremarkable. Therapy was immediately discontinued. Sputum specimens collected on 3 consecutive days were positive for acid-fast bacilli. Nucleic acid amplification assays and cultures performed on mycobacteria growth indicator tube (Bactec MGIT; Becton Dickinson, Franklin Lakes, NJ, USA) and on Lowenstein-Jensen medium were positive for *Mycobacterium tuberculosis* isolates that later showed sensitivity to streptomycin, isoniazid, rifampin, and ethambutol.

The patient was isolated at the Institute of Respiratory Diseases, University of Sassari–Faculty of Medicine, Sassari, Italy. A chest radiograph showed opacity in the upper right lung, and a high-resolution computed tomography scan (Figure) showed multiple lesions that were considered compatible with TB. CD4+ cell count (52.4%; 669 cells/mm^3^) was within reference range.

**Figure:**
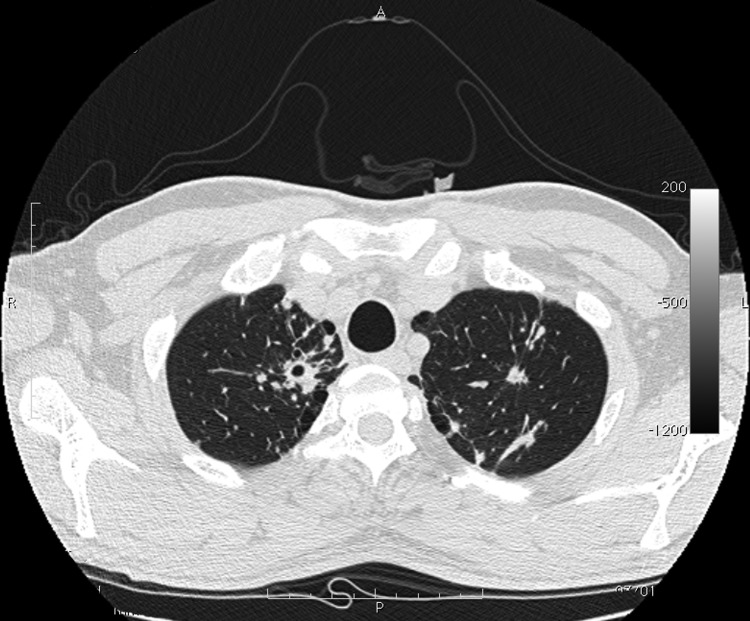
Computed tomography image of chest of patient with tuberculosis after anti–hepatitis C virus therapy. A parenchymal distortion 32 mm in diameter is shown in the upper right lung with initial central excavation 10 mm in diameter. Similar lesions 8 mm in diameter without central excavation are shown in the upper left lung.

TB treatment with rifampin, isoniazid, pyrazinamide, and ethambutol with pyridoxine was started. After 4 weeks of therapy, 3 sputum specimens were negative for acid-fast bacilli, but a bacterial culture was still positive; mycobacteria indicator growth tube culture was negative after 5 weeks.

The interaction process between the IFN-α/β system and *M. tuberculosis* is not well known; nevertheless, Peg- IFN, alone and in combination with ribavirin, is considered potentially immunosuppressive (*4**,**9*). Immunodeficiency caused by Peg-IFNs and ribavirin may cause lower leukopenia/lymphopenia values than expected during anti-HCV treatment and may also lower CD4+ cell count and function (*10*).

In the patient reported here, CD4+ cell count was within the reference range, and lung TB with excavations developed after 34 weeks of therapy. Before TB diagnosis, the patient had not shown any signs or symptoms of other infections and had not mentioned serious adverse effects from Peg-IFN and ribavirin treatment. However, the initial symptoms of TB and the common side effects of Peg-IFN therapy can be similar, which could have led to a delay in the diagnosis of TB.

In conclusion, even if only a few cases of active TB have been reported in the literature, it is well known that standard anti-HCV treatment increases the risk for infections. A high proportion of patients with positive purified protein derivative results, isolation of >30% of multidrug-resistant strains of *M. tuberculosis*, and high prevalence of HCV antibody are concomitant among inmates. These data, together with current recommendations for increasing use of Peg-IFN and ribavirin in marginalized populations in correctional facilities, show the need to consider TB risk before starting HCV antiviral therapy. The management of simultaneous HCV and *M. tuberculosis* infections in prisons presents particular difficulties and pitfalls to overcome. In prisons, the clinical history of inmates should be carefully evaluated, a tuberculin skin test or Quantiferon TB in Tube test (Cellestis, Melbourne, Australia) should be performed, and, if those results are positive, a chest radiograph should be taken. Before receiving Peg-IFN, purified protein derivative–positive patients should receive anti-TB chemoprophylaxis. The case described here underscores the need for a careful and multidisciplinary evaluation of inmate patients for latent TB before administration of Peg-IFN and ribavirin therapy, thus avoiding reactivation.
